# Effect of recombinant human erythropoietin combined with iron sucrose on postoperative hemoglobin in patients undergoing artificial joint replacement

**DOI:** 10.1038/s41598-023-41887-8

**Published:** 2023-11-02

**Authors:** Wenjiang Yu, Chengyan Liu, Zhiguo Bi

**Affiliations:** 1 Department of Orthopedics, Linyi People’s Hospital, Linyi, Shandong China; 2 Department of Bone and Joint Surgery, The Second Affiliated Hospital of Xi’an Jiaotong University, Xi’an, China; 3 Department of Orthopedics, The First Hospital of Jilin University, Changchun, Jilin China

**Keywords:** Outcomes research, Rheumatology

## Abstract

With the aging of the population, an increasing number of elderly patients are opting for artificial joint replacement, leading to the exploration of various rapid rehabilitation programs in the perioperative period. In this study, we aimed to investigate the effectiveness of combining recombinant human erythropoietin and iron sucrose in altering the range and trend of postoperative hemoglobin in patients undergoing arthroplasty. Specifically, we will examine whether this combination can effectively alter the rise and fall of postoperative haemoglobin, identify the inflection point of haemoglobin change or recovery after arthroplasty, and assess the effect of treatment on serum iron in postoperative blood. We conducted a retrospective study of 138 patients who underwent unilateral total joint arthroplasty by the same surgeon in the same hospital before July 2022. The results of this study may provide valuable insights for the development of effective rehabilitation programs for patients undergoing arthroplasty.

## Introduction

For arthroplasty, perioperative blood management is an important sector that cannot be ignored. Relevant experiments show that in the initial TKA and THA, the average bleeding volume calculated in the visible and invisible aspects is approximately 1500 ml^1,2^, and then the haemoglobin values decrease by approximately 3 g/dl^1,3^. However, with advances in surgical techniques and increased emphasis on perioperative blood management, intraoperative bleeding in joint replacement surgery is kept low and hardly affects intraoperative hemoglobin levels^4^. Moreover, total perioperative bleeding was significantly lower at 290–860 ml^5–7^. The experiment of Park et al. deemed that in patients receiving THA, multivariate analysis showed that blood loss increased with age (3.44 ml increased every year) and body mass index (12.45 ml increased every 1 kg/m^2^)^3^. Sehat et al. concluded that perioperative recessive blood loss in TKA is often more than 50% of the total blood loss^2^. In addition, it was confirmed that there are a series of complications in blood transfusion, and there is a certain connection with joint infection^8–12^. Therefore, many institutions and hospitals are studying how to reduce the total blood loss, improve the hemoglobin value, and reduce the blood transfusion rate during the perioperative period. Maempel et al. reported that the preoperative haemoglobin levels of women and men undergoing TKA were 12.75 g/dl and 13.75 g/dl, respectively, which have been identified as the lowest threshold for reducing the need for blood transfusion, with maximum comprehensive sensitivity and specificity^8^. However, preoperative optimization often requires a long treatment time, which increases various economic costs. In addition, the self-management of nonhospital patients is poor. The outcome is that it is difficult to ensure that the haemoglobin value of patients on the day before surgery reaches the threshold. Therefore, intraoperative and postoperative blood management will receive more attention.

The use of tranexamic acid in joint replacement surgery has been proven to be able to effectively reduce the amount of bleeding and reduce the intraoperative and postoperative blood transfusion rates^13–25^. The usage scheme is from one dose to six doses. Although venous thrombosis is often a concern for the side effects of tranexamic acid, any combination of these dosages has been reported not to increase the incidence or risk of postoperative deep venous thrombosis in patients undergoing arthroplasty^13,14,16–20,25^. This is significantly lower than the increased costs associated with postoperative rehabilitation, such as blood transfusion, hospital inspection, length of stay, diet and so on^26,27^, so this measure has been routinely adopted by major hospitals.

Ferrimodulin is the main systemic iron regulatory hormone of iron metabolism, and its synthesis is controlled by a variety of signaling pathways (such as inflammation, hypoxia, and erythropoietin)^28^. The high level of ferrimodulin caused by inflammation related to surgical stress will inhibit the intestinal absorption of oral iron. In addition, adverse reactions of the gastrointestinal tract after surgery are of large probability. Therefore, postoperative anemia is more common (up to 80–90%). Sometimes, high-dose oral iron therapy is not better than placebo^29–31^. Intravenous iron is similar to or better than oral iron in improving hemoglobin levels and decreasing blood transfusion rates^32^. Therefore, in the case of intraoperative or postoperative anemia, intravenous iron is preferred to oral iron. If the reaction of intravenous iron is insufficient and there is no contraindication for patients, the combination of recombinant human erythropoietin (EPO) and intravenous iron should be considered^29^. Relevant studies have shown that the addition of a single dose of rh-EPO (40,000 IU) can enhance the erythropoiesis effect of intravenous iron and further reduce the demand for blood transfusion^33^.

For the use of rh-EPO, most studies tend to give preoperative medication to patients with preoperative anemia. Usually, 30,000–40,000 IU/w or 10,000 IU/day is given subcutaneously, and the program starts 2–4 weeks before surgery. On this basis, some experiments have also used various iron agents orally or intravenously^34–40^. There are also many experiments that have verified more convenient schemes: we can use rh-EPO and intravenous iron once before or on the day after surgery and then inject rh-EPO (3000–10,000 IU) subcutaneously every day or every other day after surgery, equipping intravenous iron (100–200 mg)^41–43^. In contrast, there are individual reports suggesting that this kind of practice is not worth taking because the high cost may make it too expensive for routine use, and the effectiveness of reducing the blood transfusion rate and accelerating postoperative rehabilitation after surgery is not largely obvious^44,45^. The main purpose of this study was to verify the short-term effect of the combined use of rh-EPO and sucrose iron on postoperative haemoglobin.

## Methods

All methods were carried out in accordance with relevant guidelines and regulations. The study was performed in accordance with the Declaration of Helsinki. All experimental protocols were approved by the ethics committee of the First Hospital of Jilin University, and the need for informed consent was waived by the ethics committee of the First Hospital of Jilin University since only anonymized patient data were used for the study.

The study included adult patients (> 18 years old) who underwent total joint arthroplasty performed by the same team of orthopedic surgeons. Preoperative exclusion criteria consisted of preoperative autologous blood donation, iv iron or erythropoietin therapy; active infection; pregnancy or lactation; major comorbidities (previous history of stroke, transient ischemic attacks, or seizures); significant respiratory disease (forced expiratory volume in the first second < 50% predicted), renal disease (creatinine > 200 µmol/l], or liver disease (hepatitis, cirrhosis); uncontrolled hypertension (systolic > 180, diastolic > 100 mmHg); and any hematological diseases (e.g., thromboembolic events, hemoglobinopathy, coagulopathy, or hemolytic disease). Additionally, patients with ongoing hemorrhage or evidence of organ dysfunction on postoperative days were also excluded.

A total of 150 patients with unilateral total joint replacement performed by the same surgeon in the same hospital were selected consecutively, and the final sample size for the study was 138 (99 in the control group and 39 in the experimental group). The preoperative examination of all patients was in line with the surgical indications, and there was no surgical contraindication. In the experimental group, rh-EPO (10,000 iu) was injected subcutaneously every day; a dose of sucrose iron (200 mg) dissolved in 250 ml normal saline was administered intravenously every other day after the operation. The control group was only supported by symptomatic treatment after the operation. No drainage tube was used in any patients.

The demographic data of the two groups were similar (*p* > 0.05) and comparable (Tables 1, 2). We collected the haemoglobin and serum iron of patients who were included in the experimental study on the first, third and fifth days after the operation and calculated the difference values of haemoglobin (HB1-3, HB5-3) and serum iron (Fe3-1, Fe5-3) and then judged the normality of clinical data between the two groups. If the data were normally distributed, an independent sample t test was used. If the distribution was skewed, a rank sum test was used. We assigned 1 to the patients whose values were greater than or equal to 0 in HB1-3 and HB5-3 and 2 to the patients whose values were less than 0 to convert them into categorical variables. For categorical variables, the chi-square test was adopted. According to the trend and extent of the increase or decrease in hemoglobin value, we will evaluate the effect of the combined use of rh-EPO and sucrose iron on the rapid recovery of patients undergoing TKA or THA. At the same time, according to the change in serum iron content, we can analyse whether rh-EPO can effectively promote the optimal utilization of iron in the blood and then promote the formation of red blood cells.

**Table 1:** Comparison of demographic data of the two groups in terms of hemoglobin value (n = 138).

Group	Age (year)	Body mass index/(kg·m^2^)	Sex	
			Man	Woman
Control group (n = 99)	65.03 ± 10.08	25.77 ± 3.92	35 (35.4%)	64 (64.6%)
Experimental group (n = 39)	67.82 ± 11.36	25.03 ± 2.76	18 (46.2%)	21 (53.8%)
Difference and 95% CI	− 2.790 (− 6.698 to 1.117)	0.746 (− 0.613 to 2.105)		
Test statistics	T = − 1.412	T = 1.086	Chi square = 1.380	
p	0.160	0.279	0.240

**Table 2:** Comparison of demographic data of the two groups in terms of serum iron (n = 99).

Group	Age (year)	Body mass index/(kg·m^2^)	Sex	
			Man	Woman
Control group (n = 63)	63.97 ± 10.10	26.07 ± 3.62	23 (36.5%)	40 (63.5%)
Experimental group (n = 33)	67.55 ± 11.91	24.73 ± 2.64	15 (45.5%)	18 (54.5%)
Difference and 95% CI	− 3.577 (− 8.164 to 1.010)	0.746 (− 0.078 to 2.756)		
Test statistics	T = − 1.548	T = 1.877	Chi square = 0.725	
p	0.125	0.064	0.395	

Presurgery Hb levels and Hb measured on the day after surgery were used to calculate the Hb change due to surgery. The Nadler formula was used to calculate blood volume (BV): k1 × h^3^ + k2 × m + k3 (where h is height, unit: m, m is weight, unit: kg); k values were as follows: males—k1 = 0.366 9, k2 = 0.032 19, k3 = 0.604 1 and females—k1 = 0.3561, k2 = 0.03308, k3 = 0.1833^46^.

Meunier’s formula was used to calculate and report total blood loss: BV × [(Hb_i_ − Hb_e_)/Hb_e_] (where Hb_i_ = haemoglobin concentration preoperatively; Hb_e_ = haemoglobin concentration postoperatively)^47^.

All statistical analyses were performed using SPSS software (IBM SPSS Statistics, Version 25). Metric data are presented as the mean ± standard deviation (SD) ($\overline{{\text{x}}}$ ± s), and t tests were performed. Nominal data are expressed as interquartile and percentages (M (IQR)), and the rank sum test was performed for comparison. Enumeration data were expressed as rates, and chi-square tests were performed. The Mann‒Whitney U test was used to compare nonparametric variables, whereas the Wilcoxon signed-rank test was used to compare related samples of nonparametric data. A *p* value < 0.05 was defined as statistically significant.

## Result

In the study of hemoglobin, there were 99 patients in the control group, with average values of HB1-3 (14.07 ± 9.01) (g/l) and HB5-3 (2.99 ± 6.87) (g/l); there were 39 patients in the experimental group, with average values of HB1-3 (11.15 ± 7.27) (g/l) and HB5-3 (4.79 ± 6.63) (g/l). There was no significant difference between the two groups (T_HB3-1_ = 1.802, *p*_HB3-1_ = 0.074; T_HB5-3_ =  − 1.404, *p*_HB5-3_ = 0.064) (Table 3).

**Table 3:** Comparison of postoperative HB1-3 and HB5-3 values between the two groups.

Group	HB1-3 (g/l)	HB5-3 (g/l)
Control group (n = 99)	14.07 ± 9.01	2.99 ± 6.87
Experimental group (n = 39)	11.15 ± 7.27	4.79 ± 6.63
Difference and 95% CI	2.917 (− 0.284 to 6.117)	− 1.805 (− 4.347 to 0.737)
Test statistics	T = 1.802	T = − 1.404
p	0.074	0.064

When studying serum iron, there were 63 patients in the control group. The mean value of Fe3-1 was 0.816 ± 4.230 µmol/l, and the median value of Fe5-3 was 3.100 µmol/l (1.400, 4.900). There were 33 patients in the experimental group. The mean value of Fe3-1 was (− 14.724 ± 16.535) µmol/l, and the median value of Fe5-3 was 0.400 µmol/l (− 5.950, 2.900). There was a significant difference between the two groups (T_Fe3-1_ = 5.309, *p*_Fe3-1_ < 0.001; Z_Fe5-3_ =  − 3.183, *p*_Fe5-3_ = 0.001) (Tables 4, 5).

**Table 4:** Comparison of postoperative Fe3-1 values between the two groups.

Group	Mean ± standard deviation umol/l	Difference and 95% CI	T test	
			T value	p value
Control group (n = 63)	(0.816 ± 4.230)	15.540 (9.593–21.488)	5.309	< 0.001
Experimental group (n = 33)	(− 14.724 ± 16.535)

**Table 5:** Comparison of postoperative Fe5-3 values between the two groups.

Group	M (P25, P75) umol/l	Median of difference umol/l (95% CI)	Wilcoxon rank sum test	
			Z value	p value
Control group (n = 63)	3.100 (1.400, 4.900)	3.200 (1.300–5.500)	− 3.183	0.001
Experimental group (n = 33)	0.400 (− 5.950, 2.900)			

**Figure 1:**
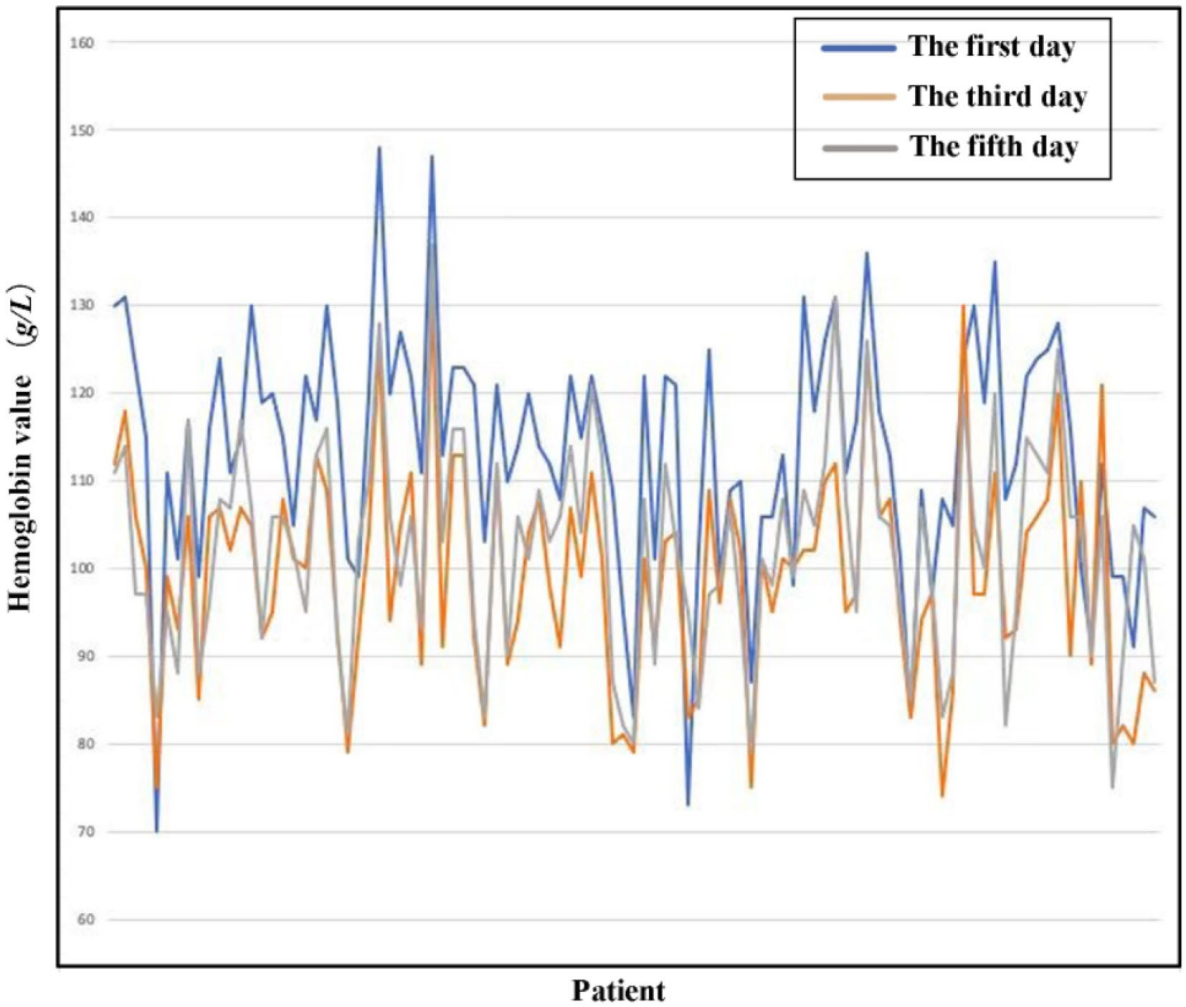
Overall comparison of postoperative hemoglobin of the control group. The vertical coordinate represents the hemoglobin value and the vertical coordinate represents the patient. And the blue line represents the first day, the orange line represents the third day, and the grey line represents the fifth day. This graph not only intuitively reflects the trend of hemoglobin change over time in the control group, but also shows the differences between individuals of different patients, suggesting the significance of individualized medical treatment.

**Figure 2:**
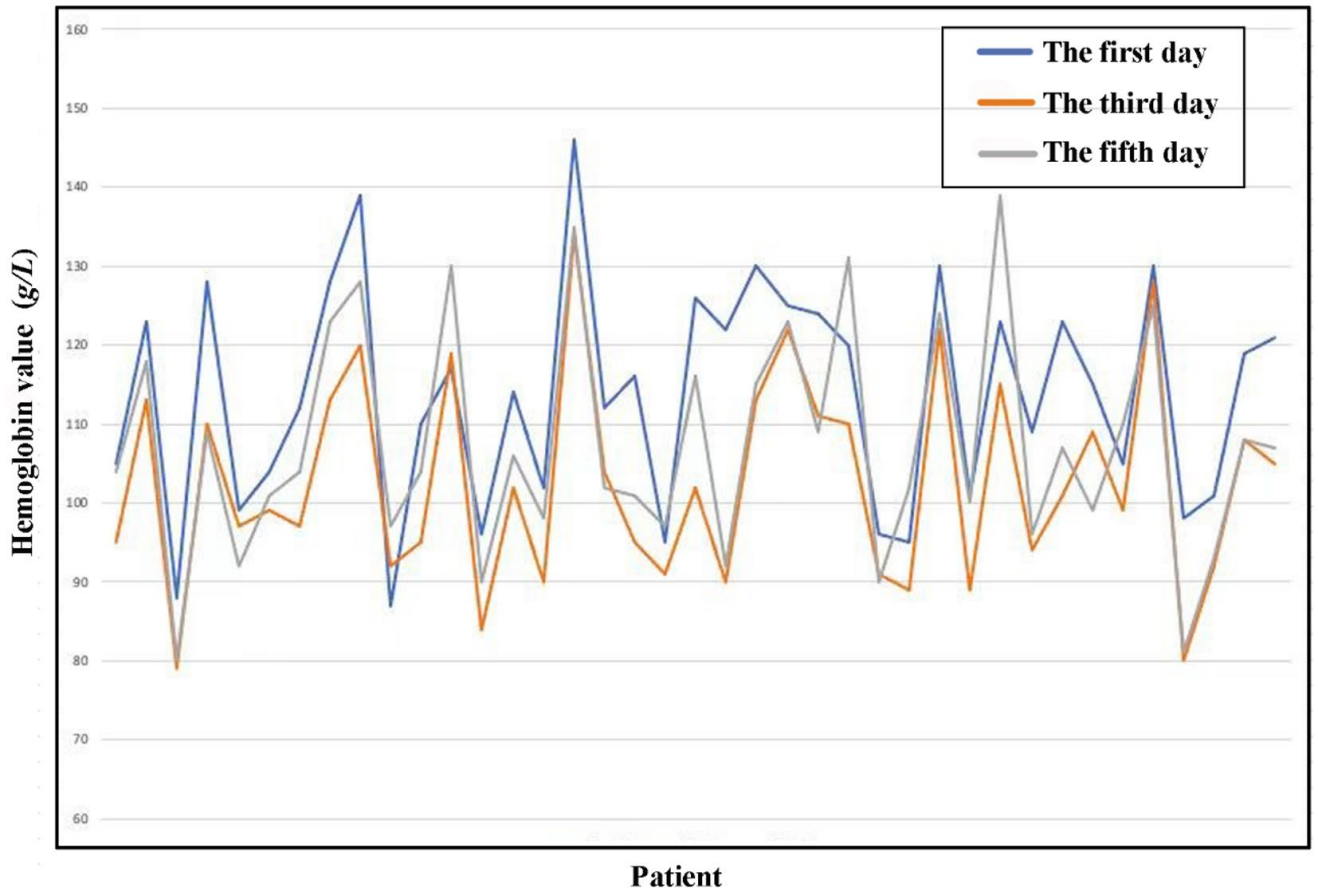
Overall comparison of postoperative hemoglobin of the experimental group. The vertical coordinate represents the hemoglobin value and the vertical coordinate represents the patient. And the blue line represents the first day, the orange line represents the third day, and the grey line represents the fifth day. This graph not only intuitively reflects the trend of hemoglobin change over time in the experimental group, but also shows the differences between individuals of different patients, suggesting the significance of individualized medical treatment.

For the change trend of hemoglobin, the data show that for HB1-3, the proportion of the control group assigned 1 is 93.9%, and the proportion of the experimental group assigned 1 is 94.9% (chi square value < 0.0001, *p* = 1). For HB5-3, 72.7% of the control group and 82.1% of the experimental group are assigned 1 (chi square = 1.310,* p* = 0.252). There was no significant difference between the two groups. It can be concluded from the data that from the first day to the third day after surgery, the haemoglobin of the experimental group and the control group generally showed a downwards trend, and from the third day to the fifth day after surgery, the haemoglobin of the patients in the two groups generally tended to be stable or upwards (Tables 6, 7). The results shown in the data plot of all patients are basically the same as above. The hemoglobin values of most patients on the first and fifth days after surgery were greater than those on the third day (Figs. 1, 2).

**Table 6:** Comparison of postoperative hemoglobin trends (HB1-3) between the two groups.

Group	Total	1 (number (%))	2 (number (%))	Chi square test	
				Chi square	p value
Control group	99	93 (93.9%)	6 (6.1%)	< 0.0001	1
Experimental group	39	37 (94.9%)	2 (5.1%)

**Table 7:** Comparison of postoperative hemoglobin trends (HB5-3) between the two groups.

Group	Total	1 (number (%))	2 (number (%))	Chi square test	
				Chi square	p value
Control group	99	72 (72.7%)	27 (27.3%)	1.310	0.252
Experimental group	39	32 (82.1%)	7 (17.9%)

There was no statistically significant difference in the amount of perioperative blood loss over time between the control and experimental groups (*p* = 0.95 and 0.091, respectively), as depicted in Table 8.

**Table 8:** Comparison of blood loss between the two groups.

Group	The third day (ml)	The fifth day (ml)
Control group	616.32 ± 433.06	174.28 ± 242.74
Experimental group	483.72 ± 373.17	109.51 ± 267.49
Difference and 95% CI	132.60 (− 23.38 to 288.58)	− 64.77 (− 162.28 to 32.74)
Test statistics	T = 1.681	T = − 1.314
*p*	0.095	0.191

The third day represents the amount of blood lost in the surgical patient over 3 days compared to the preoperative period. The fifth day represents the amount of blood lost in 3 days compared to the third through fifth postoperative days.

The data on adverse events after the use of the drug are listed in Table 9. Upon assessing the correlation between the complications and drug utilization, there was no statistical significance for deep vein thrombosis (6.1% vs 7.7%, *p* = 0.727), gastrointestinal symptoms (12.1% vs 17.9%, *p* = 0.371) and fever (17.1% vs 20.5%, *p* = 0.528). None of the patients had allergic reactions, pulmonary embolisms, periprosthetic fractures, or wound infections postoperatively.

**Table 9:** Adverse events of the two groups of patients after the use of the drug.

Group	Control group	Experimental group	Z/χ^2^	*p*
DVT	6 (6.1%)	3 (7.7%)	0.122	0.727
Gastrointestinal symptoms	12 (12.1%)	7 (17.9%)	0.800	0.371
Fever	17 (17.1%)	8 (20.5%)	0.432	0.528
Allergic reaction	0	0		
Pulmonary embolism	0	0		
Periprosthetic fracture	0	0		
Wound infection	0	0		

*DVT* deep vein thrombosis.

*p < 0.05 compared with the preoperative values.

## Discussion

In recent years, the perioperative blood transfusion rate of arthroplasty has decreased yearly, which benefits from gradual attention to perioperative blood management and the update and progress of knowledge^48^. There is a certain relationship between blood transfusion and joint infection, and joint infection can be said to be a catastrophic complication for such surgery^8–12^. Therefore, many institutions and hospitals are studying how to reduce the blood transfusion rate. Hemoglobin value is one of the most important indications of whether blood transfusion is needed.

Long-term preoperative intervention programs have been proven to be effective in reducing the blood transfusion rate and rapid recovery after surgery, but their controllability and compliance are poor and cannot be generally implemented^34–40^. Patients are now generally willing to accept intraoperative and postoperative blood management programs. The use of different doses of tranexamic acid on the day of operation has been proven to be able to effectively reduce the amount of bleeding caused by the operation to reduce the rate of blood transfusion^13–25^. Similarly, some experimental studies claim that the combined use of rh-EPO and iron agents can have an effective effect in a short period of time^41–43^, but such data do not seem to be completely consistent with our conclusions.

In our retrospective study, the mean values of HB1-3 in the two groups were positive, and the difference was approximately 2.9 g/l (within the 95% confidence interval), which means that the hemoglobin values of the two groups were declining, and the declined value of the patients in the experimental group was smaller than that of the control group. It can be roughly believed that the combined use of these two types of drugs has a certain effect on improving the haemoglobin value in the short term to some extent. However, such a small change has no significant impact on the overall change amplitude, and there is no significant difference (THB3-1 = 1.802, PHB3-1 = 0.074). The same is true for HB5-3. From the perspective of the change trend of the whole haemoglobin value, there was no significant difference between the two groups. The hemoglobin values of the two groups of patients were basically in the decline stage in the first 3 days after surgery and rose or basically remained the same 3 days later (chi square value < 0.0001, p = 1).

The reason why the results are different from those obtained in other articles may be as follows: First, some articles research the numerical changes on the day of operation and the first 3 days after operation. They believed that more than 80% of the total blood loss often occurs within 24 h after the operation^41^. Some experiments also showed that the decrease in haemoglobin within 1 day after the operation was the largest^42^. Our experiment was performed to study the numerical changes on the first, third and fifth days after surgery. At this stage, perhaps the hemoglobin values in the human body have become relatively stable without large fluctuations, so even if these two drugs are used, the values will not float significantly. Second, the method of using tranexamic acid during the operation of patients in this experiment is to apply a dose of tranexamic acid (20 mg/kg) half an hour before the beginning. If the surgery lasted more than three hours, another dose of tranexamic acid was applied; otherwise, it was not administered. However, the scheme in the related article is to inject two doses of tranexamic acid (every 3 h)^42^. Whether the difference in the use of tranexamic acid will affect the experimental results remains to be investigated. Third, many researchers started using rh-EPO and iron more than 2 weeks before surgery. They believed that although rh-EPO could promote the use of iron and the production of red blood cells, it could not rapidly and significantly increase hemoglobin in a short time^29,33–40^. Whether our experimental research is too short to observe the real effect also needs to be further explored. Since our research shows that hemoglobin begins to rise or stabilize three days after surgery, it is meaningless that these drugs have an effective impact even on the fourth day.

Reducing the blood transfusion rate is the ultimate objective of most experiments. In our study, we mainly discussed the impact on hemoglobin. Although the impact is small and not statistically significant, small changes here may have a greater influence on whether blood transfusion is needed. Strict adherence to appropriate transfusion standards often has a huge effect on the results of the study. In postoperative patients, when the standard for triggering blood transfusion was set as a hemoglobin value lower than 7–8 g/dl rather than a more relaxed threshold (9 or 10 g/dl), no difference was found^49–52^. A meta-analysis of relevant experiments showed that the use of restrictive transfusion strategies with hemoglobin values less than 7 g/dl could significantly reduce various complications, such as acute coronary syndrome, pulmonary edema, rebleeding, infection, and total mortality, compared with more liberal strategies. There may also be clinical conditions requiring blood transfusion when the hemoglobin value is higher than 7–8 g/dl or even higher. The main signs include hypotension, tachycardia, shortness of breath, dizziness, fatigue, etc. In the case of the above situations, a blood transfusion scheme is required when necessary^52–55^. Because our control of the blood transfusion standard is not strict enough and this study is aimed at the analysis of the range and trend of the value changes from the first day to the fifth day after surgery, we do not consider whether blood transfusion was implemented during surgery as one of the influencing factors. In contrast, we excluded all patients who received blood transfusion during surgery to avoid affecting the data analysis.

According to the collected serum iron data, during the five days of hospitalization after surgery, the rise and fall of serum iron in patients in the control group were significantly weaker than those in the experimental group, and there was a significant difference between the two groups (TFe3-1 = 5.309, PFe3-1 < 0.001; ZFe5-3 =  − 3.183, PFe5-3 = 0.001). Theoretically, compared with the control group, the serum iron in the human body tends to increase significantly in a short period of time after the supplementation of iron, but in the case of our retrospective study, the serum iron of the patients in the experimental group decreased evidently. Based on the above research data and phenomena, we can conclude that the use of rhEPO will accelerate the redistribution and utilization of iron ions in the body, which will reduce the content of iron ions in the blood.

The Meunier formula is widely used to calculate postoperative blood loss and is particularly applicable for calculating transfusion risk^47,56,57^. The present study found that the combined use of rhEPO and iron source in the short term did not reduce perioperative blood loss, which is consistent with the results of other studies^58^. The potential causes are that up to 10% of patients exhibit EPO resistance or hyporesponsiveness^59^. In addition, factors may be more significant drivers of perioperative blood loss, such as extensive soft tissue release and bone cuts in joint arthroplasty^60^, use of anticoagulants^61^, and individual coagulation factors^62^.

The relatively mild adverse events associated with rhEPO administration include a local rash, fever, edema, and constipation^63^, which will resolve gradually over time. Meanwhile, the incidence of hypertension with erythropoietin use is estimated to be approximately 10% to 15% and is associated with NO, endothelins, and the sympathoadrenal and renin-angiotensin pathways^64,65^. Nevertheless, no abnormal hypertension was found in this study because rhEPO-induced hypertension may not occur in the short term^66^. In addition, several studies have shown that rhEPO can cause severe adverse effects, such as cerebral hemorrhage and thrombosis, suggesting that rhEPO has tumor-promoting ability^67^. However, the adverse events among the patients in this study were mainly DVT, gastrointestinal reactions and fever. The incidence of adverse events was not statistically significant compared to the control group, which is similar to the results of previous studies^58^. This may be due to the strict control of preoperative patients’ physiological indexes, strict control of the indications for drug use, and timely detection and management of patients’ preexisting symptoms. Therefore, it is important to formulate appropriate individualized therapeutic measures when treating patients with rhEPO combined with iron and to pay timely attention to patients’ postoperative status to prevent the occurrence of serious complications.

We learned from the relevant literature that the use of tranexamic acid on the day of operation with no more than six doses (6 g in total, intravenous once every three hours) had no effect on the probability of postoperative deep vein thrombosis^13,14,16–20,25^, and it was feasible to reduce the probability of blood transfusion in hospitals^13–25^. Based on this and the change trend of hemoglobin value obtained by our research, a scheme seems to achieve the maximum optimization effect: we can only use rh-EPO (10,000 iu) and iron sucrose (200 mg) together during the operation and the first 3 days after the operation (once a day). In addition to the two doses of tranexamic acid on the day of operation, we can take a daily intravenous drip (1 g/day) in the first 3 days after the operation. In addition, according to our research results, the average value of HB1-3 is 14.07 ± 9.01 g/l, and if the hemoglobin value on the first day after surgery is greater than 90 g/l, we can choose not to take any intervention measures.

## Conclusion

In conclusion, the combined use of rh-EPO and iron sucrose after surgery can accelerate the redistribution and utilization of iron ions in the body in a short period of time, but there is no statistical significance for the change in hemoglobin value, and it cannot advance the rebound of hemoglobin. Therefore, it not only cannot play a greater role in the postoperative rehabilitation of patients undergoing arthroplasty but also incurs additional medical costs.

## Data Availability

The datasets used and analysed during the current study are available from the corresponding author on reasonable request.
