# Author Correction: Effect of recombinant human erythropoietin combined with iron sucrose on postoperative hemoglobin in patients undergoing artificial joint replacement

**DOI:** 10.1038/s41598-023-48937-1

**Published:** 2023-12-29

**Authors:** Wenjiang Yu, Chengyan Liu, Zhiguo Bi

**Affiliations:** 1 Department of Orthopedics, Linyi People’s Hospital, Linyi, Shandong China; 2 Department of Bone and Joint Surgery, The Second Affiliated Hospital of Xi’an Jiaotong University, Xi’an, China; 3 Department of Orthopedics, The First Hospital of Jilin University, Changchun, Jilin China

Correction to: *Scientific Reports* 10.1038/s41598-023-41887-8, published online 02 November 2023

The original version of this Article contained errors in the Affiliations.

Wenjiang Yu was incorrectly affiliated with ‘Department of Orthopedics, The First Hospital of Jilin University, Changchun, Jilinx, China’.

In addition, Chengyan Liu was incorrectly affiliated with ‘Department of Orthopedics, Linyi People’s Hospital, Linyi, Shandong, China’.

Finally, Zhiguo Bi was incorrectly affiliated with ‘Department of Orthopedics, Linyi People’s Hospital, Linyi, Shandong, China’. The correct affiliation for Zhiguo Bi is listed below.

Department of Orthopedics, The First Hospital of Jilin University, Changchun, Jilin, China.

As a result, the Affiliations have been renumbered.

The original Article has been corrected.

